# The relationship between depression and risk of violence in portuguese community-dwelling older people

**DOI:** 10.1186/s12889-022-13474-5

**Published:** 2022-06-06

**Authors:** Felismina Mendes, Joana Pereira, Otília Zangão, Catarina Pereira, Jorge Bravo

**Affiliations:** 1 Escola Superior de Enfermagem São João de Deus, Universidade de Évora, Largo do Sr. da Pobreza 2B, Évora, Évora Portugal; 2 Comprehensive Health Research Centre (CHRC), Universidade de Évora, Largo dos Colegiais 2, Évora, Portugal; 3 Departamento de Desporto e Saúde, Escola de Saúde e Desenvolvimento Humano, Universidade de Évora, Largo dos Colegiais 2, Évora, Portugal

**Keywords:** Depression, Non-institutionalized, Older adults, Risk factors, Violence prevention

## Abstract

**Background:**

Mental disorders are highly prevalent in older people, being depression a predominant disorder. Evidence points to a possible relationship between depression and violence against older people. Nonetheless, the role of the depressive symptomology severity in the risk of violence against older people remains unclear. Thus, this study’s main objective was to analyze the relationship between geriatric depressive symptomatology and the risk of violence against older people.

**Methods:**

This exploratory study involved 502 community-dwelling older persons aged 65 to 96 years (73.3 ± 6.5). Measures were performed using the Geriatric Depression Scale and the Risk Assessment of Violence against the Non-Institutionalized Elderly scale.

**Results:**

One hundred nineteen older people (23.7%) had mild/moderate depressive symptomology, and twenty-six (5.2%) had severe depressive symptomology. There were significant relationships between the severity of depressive symptomatology and the risk of violence (*p* < 0.05). The presence of depressive symptomatology increased the likelihood of being victims of violence, particularly among women (odds ratio: 2–8, *p* < 0.05).

**Conclusions:**

The severity of depressive symptomatology plays an essential role in the risk of violence against community-dwelling older people. Moreover, it was found that older persons with depression symptomatology were at higher risk of being victims of violence. Our study findings support the need for protective measures within mental health national or regional policies to prevent depression and violence against community-dwelling older people.

## Background

Life expectancy has increased in recent decades [1]. In 2015, Portugal had a longevity index of 48.8% and a global aging index of 148.7%. In the Alentejo region, the aging index was even higher, namely was of 193.1% [2]. These Portuguese population aging indicators place Portugal among the oldest countries globally [3].

According to the literature, there is a high prevalence of mental disorders, and depression predominates in older people [4]. Depression prevalence in community-based older people vastly differs worldwide, varying between 17.6% in the United Kingdom and 33.5% in Japan [5, 6], depending on screening tools and survey samples. A recent study performed with Portuguese older people found that 15.3% of the participants were severely depressed. In addition, 55.9% had an on-growing severity of depression [7].

In older people, depressive symptomatology is usually related to loss of pairs and relationships, loss of family and occupational roles, low economic resources, physical health deterioration, weak social support networks and loneliness [8–11]. Disability, decreased functional capacity and quality of life [8] are examples of the negative consequences of this health condition. Moreover, depressive symptomatology in older people emerges at a stage of life in which higher difficulties related to chronic diseases (comorbidity and multimorbidity) arise, directly impacting the loss of autonomy and isolation [8].

Violence against older people is a multidimensional public health problem that may be described as “a single or repeated act, or lack of appropriate action, occurring within any relationship where there is an expectation of trust, which causes harm or distress to an older person” [12]. It may occur both in community and institutional settings, perpetrated by family members, informal and formal caregivers, or acquaintances, manifested in psychological, physical, sexual, and financial violence and neglect [13]. According to a recent systematic review and meta-analysis, approximately one-sixth of community setting older people suffered from violence in the last year. Most of them reported being victims of psychological violence (11.6%) [14]. Evidenced risk factors for violence against older people include victims’ sociodemographic (ethnicity and lower education), health-related (multimorbidity, depression, alcohol abuse, and incontinence), physical (functional impairments and frailty), and psychological characteristics (cognitive impairment, loneliness, and aggressive behavior) [15, 16]. However, many older people who were victims of violence refuse to report the event, hindering the true extent and the major causes for this issue and precluding adequate community interventions [17].

Although some studies have highlighted a possible relationship between depression and violence against older people [18, 19], the role of the depressive symptomology severity in the risk of violence against older people remains unclear. We have hypothesized that studying the relationship between the depressive symptomology severity and the risk factors for violence against older people would be an important contribution to this matter. Identifying a possible relationship and understanding how different individual or social factors interact in older people victims of violence is central. Not only for the early determination of the risk for depression and violence but also for the success of the professional’s daily efforts to prevent depression and violence. Thus, the present study aims to analyze the relationship between the severity of depressive symptomatology and the risk of violence against older people.

## Methods

### Participants

The present study was conducted with community-dwelling older people (autonomous and independent) from the region of Alentejo (Portugal) within the framework of the ESACA project (Ageing Safety in Alentejo - Understanding for action). The participants who volunteered to participate in this study were recruited from senior universities, parishes, city halls, and senior associations through pamphlets distribution. The sample size was estimated through an online software as 384 (OpenEpi), keeping the confidence interval (CI) at 95% and the significance level at 5%. At the end of data collection, the eligible older adults were 517. Inclusion criteria were age ≥ 65 years old, living independently at home and the absence of severe cognitive impairments that may affect the understanding of the questionnaires (≤ 24 points on the Mini-Mental State Examination questionnaire) [20]. Fifteen potential participants were excluded: ten were aged less than 65 years, three showed a cognitive decline, and one had suffered recent health conditions resulting in a temporary loss of physical fitness or dependence. The sample for this study consisted of 502 participants aged 65 to 96 years (73.3 ± 6.5).

### Data collection

Data collection occurred between April and July 2017 at the Nursing School Laboratory of Gerontology-Psychomotor Science of the University of Évora. The collection was performed by a team of researchers trained for this purpose and familiar with the research protocol. The researchers were graduated in nursing or sports and health sciences. As many participants could not read or write because they had never attended school, each questionnaire used in the present study was completed by a researcher through an interview with all participants.

This study was approved by the University of Évora Ethics Committee for research in human health and well-being (reference number 16 − 012) and was performed following the Declaration of Helsinki. All participants provided written informed consent.

### Outcome measures

The risk of violence was assessed through the Risk Assessment of Violence against the Noninstitutionalized Elderly Scale (ARVINI) [21]. ARVINI comprises 27 questions with the response options “yes”, “no”, and “no answer”. The “yes” and “no” options were scored “1” or “0”, respectively. The 27 items aim to identify the risk of violence based on questions about the support network and social isolation, family context, cognitive and emotional difficulties and financial issues; these issues correspond to the four dimensions of violence included in the World Health Organization definition [22] (physical, psychological, sexual and financial violence), not including neglect. The total score was obtained by summing each item’s values. Higher scores indicate a greater risk of violence for the older person. The preliminary results of this scale were considered adequate in terms of reliability. This scale revealed a Cronbach’s alpha coefficient of 0.916, proving its internal consistency. Moreover, the cut-off point that provides the maximum sensitivity and specificity for predicting the risk of violence against older people was 4.5 [21].

The Portuguese version of the Geriatric Depression Scale 15 (GDS-15) was used to assess the severity of depressive symptomatology [23]. The GDS-15 score ranges from a minimum of 0 points to a maximum of 15 points, and as higher the score as higher is the depressive symptomatology. GDS-15 score may be classified as no depression (score 0 to 4), mild and moderate depression (5 to 11) or severe depression (score 12 to 15) [24]. For data analysis, it was also considered the presence (score ≥ 5) and absence of depressive symptomatology (score ≤ 4).

Sociodemographic characteristics such as age, gender and marital status were assessed through a questionnaire.

### Statistical analysis

Data analysis was performed using IBM SPSS Statistics version 24 software with descriptive and inferential statistical methods. Descriptive statistical analysis was performed, and the results were presented as absolute (number) and relative (percentage) values. Subsequently, inferential statistical analyses were performed. A chi-square test was used to analyze the relationship between depression level (no depression, mild depression, and severe depression) and each risk of violence predictor. The likelihood of depressed older people being at risk of violence was analyzed through the calculation of the odds ratio (OR) and respective 95.0% confidence interval (CI). The level of significance was set at *p* < 0.05.

## Results

Data analysis showed that of the 502 community-dwelling older people living independently and autonomously in the community who participated in this project, 390 (77.7%) participants were female, and 112 (22.3%) were male. Regarding depressive symptomatology, 361 (71.9%) had no depressive symptomatology, 114 (22.7%) had mild/moderate depressive symptomatology, and 27 (5.4%) had severe depressive symptomatology (Table 1).

**Table 1:** Depression status prevalence among participants (absolute and relative prevalence)

Depression status	Absolute prevalence (n)	Relative prevalence (%)
No depressive symptomatology	361	71.9
Mild/moderate depressive symptomatology	114	22.7
Severe depressive symptomatology	27	5.4
Total sample	502	100.0

Regarding the relationship between the severity of depressive symptomatology and the risk of violence predictors, statistically significant associations (*p* < 0.05) were found for 18 of the 27 items on the ARVINI scale, namely the ones shown in Table 2. In the main variables, increased severity of depressive symptomatology was associated with a higher prevalence of the ARVINI item category, indicating an increased risk of violence.

**Table 2:** Association between the depressive symptomatology severity and the risk of violence in older people

Items on the ARVINI Scale	No depressive symptomatology *n* (%)	Mild/moderate depressive symptomatology *n* (%)	Severe depressive symptomatology *n* (%)	X^2^	*P*-value ^a^
1 - Do you often feel alone? (Yes)	93 (46.7%)	83 (42.1%)	22 (11.2%)	99.297	< 0.001
2 - Is there someone who keeps you company on a daily basis? (No)	293 (77.9%)	73 (19.4%)	10 (2.7%)	42.644	< 0.001
3 - Is there someone who takes you shopping when you need to go? (No)	322 (73.9%)	99 (22.7%)	16 (3.7%)	27.221	< 0.001
4 - Is there someone who takes you to the doctor when necessary? (No)	324 (72.8%)	100 (22.5%)	21 (4.7%)	7.944	0.019
5 - Do you meet with friends/colleagues weekly? (No)	322 (73.9%)	95 (21.8%)	19 (30.8%)	16.248	< 0.001
6 - Do you meet with family members on a weekly basis? (No)	298 (74.3%)	86 (21.4%)	17 (4.2%)	12.569	0.002
8 – Has someone told you that you cause them a lot of/too much work? (Yes)	5 (45.5%)	3 (27.3%)	3 (27.3%)	10.680	0.005
10 - Do you feel that no one wants to be with you? (Yes)	10 (47.6%)	6 (28.6%)	5 (23.8%)	15.261	< 0.001
11 - Are you afraid of someone in your family? (Yes)	9 (40.9%)	10 (45.5%)	3 (13.6%)	10.659	0.005
13 - Has any member of your family shouted at you and called you names, making you feel ashamed? (Yes)	43 (51.8%)	30 (36.1%)	10 (12.0%)	20.425	< 0.001
14 - Has anyone in your family physically assaulted you (pushed you, hit you)? (Yes)	14 (50.0%)	8 (28.6%)	6 (21.4%)	15.955	< 0.001
15 - Has anyone in your family told you that you are sick when you know you are not? (Yes)	9 (45.0%)	7 (35.0%)	4 (20.0%)	11.240	0.004
17 - Has someone in your family taken away things that belong to you without your consent? (Yes)	17 (51.5%)	11 (33.3%)	5 (15.2%)	9.388	0.009
22 - Do you feel that other people are unfair to you? (Yes)	68 (60.2%)	35 (31.0%)	10 (8.8%)	9.462	0.009
23 - Do you have difficulty making decisions about your life? (Yes)	55 (50.5%)	45 (41.3%)	8 (8.3%)	29.784	< 0.001
24 - Do you often feel anxious/impatient? (Yes)	118 (51.5%)	88 (38.4%)	23 (10.0%)	83.010	< 0.001
25 - Do you often get irritated? (Yes)	107 (54.3%)	69 (35.0%)	21 (10.7%)	49.814	< 0.001
26 - Can you pay your bills with your income? (No)	343 (72.5%)	106 (22.4%)	24 (5.1%)	10.614	0.005

^a^* p-*value of χ2 test

The remaining nine ARVINI scale items were not significantly related to the severity of the depressive symptomatology (Items: “7 - Do you have hostile relationships with neighbors?”; “9 - Has anyone forced you to have sex against your will?”; “12 - Do you feel that no one in your family wants to spend time with you?”; “16 - Has anyone in your family forced you to do things you did not want to do?”; “18 - Has anyone in your family forced you to sign papers against your will?”; “19 - Do you trust most people in your family?”; “2’ - Does anyone in your family have problems with alcoholism?”; “21 - Does anyone in your family use drugs?”; and “27 - Can you buy food or other necessities with your income?”). In consequence, these nine items were excluded from the odds ratio analysis.

Table 3 shows the likelihood of depressed older people being at risk of violence. This table presents the odds ratio calculated for each of the 18 items on the ARVINI scale, which has shown to be related to depression (mild/moderate and severe symptomatology).

**Table 3:** Association between depressive symptomatology and the risk of violence in older people

ARVINI Scale Items	Depressive symptomatology *n* = 141 (28.7%)	OR 95% CI	*P*-value
1 - Do you often feel alone? (Yes)	105 (75%)	8.413 (5.362–13.201)	0.001
2 - Is there someone who keeps you company on a daily basis? (No)	83 (58.9%)	3.530 (2.278–5.470)	0.001
3 - Is there someone who takes you shopping when you need to go? (No)	115 (81.6%)	2.510 (1.419–4.44)1	0.001
4 - Is there someone who takes you to the doctor when necessary? (No)	121 (85.8%)	2.060 (1.109–3.826)	0.020
5 - Do you meet with friends/colleagues weekly? (No)	114 (80.9%)	2.724 (1.540–1240)	0.001
6 - Do you meet with family members on a weekly basis? (No)	103 (73%)	2.156 (1.339–3.470)	0.001
8 - Has someone told you that you cause them a lot of/too much work? (Yes)	6 (4.3%)	3.054 (0.917–10.175)	0.047
10 - Do you feel that no one wants to be with you? (Yes)	11 (7.8%)	2.860 (1.186–6.895)	0.015
11 - Are you afraid of someone in your family? (Yes)	13 (9.2%)	3.848 (1.606–9.220)	0.001
13 - Has any member of your family shouted at you and called you names, making you feel ashamed? (Yes)	40 (28.6%)	2.847 (1.751–4.629)	0.001
14 - Has anyone in your family physically assaulted you (pushed you, hit you…)? (Yes)	14 (9.9%)	2.646 (1.227–5.705)	0.010
15 - Has anyone in your family told you that you are sick when you know you are not? (Yes)	11 (7.8%)	3.206 (1.299–7.916)	0.008
17 - Has someone in your family taken away things that belong to you without your consent? (Yes)	16 (11.3%)	2.492 (1.222–5.085)	0.010
22 - Do you feel that other people are unfair to you? (Yes)	45 (32.1%)	1.957 (1.257–3.047)	0.003
23 - Do you have difficulty making decisions about your life? (Yes)	54 (38.3%)	3.307 (2.118–5.161)	0.001
24 - Do you often feel anxious/impatient? (Yes)	111 (79.3%)	7.461 (4.686–11.879)	0.001
25 - Do you often get irritated? (Yes)	90 (64.3%)	4.088 (2.703–6.183)	0.001
26 - Can you pay your bills with your income? (No)	130 (92.2%)	4.146 (1.573–10.925)	0.002

*ARVINI* Risk Assessment of Violence against the Noninstitutionalized Elderly Scale, *OR* Odds Ratio, *CI* Confidence Intervals

The odds ratio analysis revealed a statistically significant association between depression in older people and the risk of violence (*p* < 0.05), such as the likelihood of being a victim of violence was from two to eight times higher in those participants who have mild/moderate and severe depressive symptomatology than in those without depressive symptomatology (OR = 1.957 and OR = 8.413).

## Discussion

Our study results showed significant associations between the severity of depressive symptomatology and the risk of violence against community-dwelling older people. These results confirm that the higher severity of depressive symptomatology is related to an increased risk of older people being victims of violence. Furthermore, it was found that older people with depressive symptomatology were more susceptible to being at risk of violence, showing an increased likelihood of being victims of violence from two to eight times compared to those without depressive symptomatology.

According to our participant’s report, higher levels of depressive symptomatology were related to a higher risk of violence occurrence. To the authors’ knowledge, no research has studied the relationship between different levels of depressive symptomatology assessed through the GDS with violence against community-dwelling older people. Nevertheless, a similar approach examined the association between multiple dimensions of depressive symptoms and elder abuse subtypes [25]. Roepke-Buehler and colleagues (2015) found differential associations between depression dimensions and elder abuse subtypes in their study. In addition, Roepke-Buehler and colleagues (2015) concluded that older adults reporting higher levels of depressed affect, somatic complaints, and interpersonal problems were more likely to be victims of violence.

Furthermore, previous research shows that general emotional and affective support and family solidarity might be more important than instrumental support in preventing psychological depression and anxiety symptoms in older people [26]. Social isolation and the absence of a social support network are risk factors for violence against older persons, emphasizing the association between psychological and emotional violence [27]. Adverse economic, social, and health conditions are recognized to increase the severity of depressive symptoms and the overall risk of developing depression [8–11, 28] and, in consequence, leave the older person more susceptible to being a victim of violence [29, 30].

Moreover, in the present study, older people with depressive symptomatology were up to eight times more likely to experience violence than those without symptomatology. These results align with previous studies, which reported that depression is a risk factor for violence against community-dwelling older people [19, 25, 31]. Other studies reinforce that mental health problems contribute to developing or maintaining other risk factors for violence against older people, such as the provocative behavior typical in dementia patients, which is a known risk factor for psychological violence [32]. Mental health impairments may also create unrealistic expectations of older people’s capabilities and limit the perpetrator’s emotions, increasing the risk for violence episodes [33]. Similarly, older people with depressive symptomatology and higher levels of irritability and negativism may contribute to violence episodes, probably due to the high burden they cause on their partners [34]. On the other hand, violence causes physical and psychological harm, often resulting in a continued state of fear and heightened stress [22, 35], contributing to depressive symptoms growing in the older people victim of violence. Despite the contribution of our study, the analysis results do not allow us to say whether it is depression that is the origin of violence or if it is the violence that is the origin of depression. In sum, that is a consensus that violence is associated with an increased risk of depression, whereas depression is consistently associated with elder abuse [19].

Future insights may be added regarding depression and violence prevention. Health professionals and the social sector are pivotal in detecting depression and violence and providing training and education for older people and their partners or caregivers. In their care practices, health professionals and the social sector should routinely use tested tools that systematically detect depression and violence against older people - as was performed in the present study - and act promptly based on the outcomes. Additionally, particular attention should be paid to the family context when considering preventive strategies. Some studies suggested that social support may be a preventive strategy for the harm caused by violence against older people due to its significant association with quality of life and psychological well-being [36]. Favorable social support decreases the likelihood of violent episodes, acting as a buffer against stress, depression, and health problems [33]. Community active aging and well-being programs to fight social isolation and loneliness may be crucial to achieving these ends [37–39].

Some limitations shall be disclosed in this study. The proportion of male participants in the present study was widely lower than that of female participants, which limited gender comparisons. However, gender proportions in most studies are unbalanced with a higher representation in their sample, as was the case of our sample [40]. Another limitation of our study is that being an observational study does not allow establishing cause-effect relationships. Thus, it is not clear if depressive symptomatology promotes violence against older people or if the occurrence of violence promotes the development of depressive symptomatology. Nonetheless, this issue may not be crucial, such as the present study findings align with the suggestion that preventive intervention programs shall be designed to achieve both.

## Conclusions

The severity of depressive symptomatology was found to play an essential role in the risk of violence against community-dwelling older people. Generally, the higher the severity of depressive symptomatology, the higher the risk of violence. Furthermore, older persons with depressive symptomatology were at higher risk of violence. This knowledge supports the need for protective measures within mental health national or regional policies for preventing both depression and violence against community-dwelling older people.

## Data Availability

The datasets used and/or analyzed during the current study are available from the corresponding author upon reasonable request.

## Abbreviations

ESACA: Ageing Safety in Alentejo - Understanding for action
ARVINI: Risk Assessment of Violence against the Noninstitutionalized Elderly Scale
GDS-15: Geriatric Depression Scale 15
IBM: International Business Machines Corporation
SPSS: Statistical Package for the Social Sciences
OR: Odds Ratio
CI: Confidence Interval
